# Epidemic Typhus Fever in Ohio

**Published:** 1844-09

**Authors:** John Dawson

**Affiliations:** Jamestown, Ohio


### WESTERN JOURNAL

OF

### MEDICINE AND SURGERY.

SEPTEMBER, 1844.

ART. I.—*Epidemic Typhus Fever in Ohio.* By JOHN DAWSON, M.D., of Jamestown, Ohio.

The object of this paper is to lay before the profession some facts in regard to an Epidemic Typhus Fever, as it manifested itself in some twenty-one cases, that have occurred of late in my practice.

Nineteen of these cases took place in persons of middle age. Of the remaining two, one was sixty years old, and the other was fifteen.

Sixteen of the cases occurred in males, generally of but a tolerable habit of body; and five in females, two of which labored under a nervous diathesis, while the remaining three possessed an ordinary share of constitutional vigor.

Five of the cases were of spontaneous origin, occurring in persons who had not been exposed to any known sources of contagion. The others were able to trace the causes of their indisposition to attention upon those already laboring under the disease.

*Complications.*—Under this head we hardly know how to write, for so various have been the views, as we shall subsequently see, of typhus fever, not only in ancient, but to some extent in modern times, that it is next to an impossibility to consider any thing as a complication, that has not, at some period or other, been urged as a part of the disease itself. Valid, hower, as these objections may be, for the sake of convenience we will speak of the phenomena presented in these cases as complications

With hemorrhage of the bowels two cases were complicated. The hemorrhage took place in one about the fourteenth day, in the other it occurred on the twenty-first day. The quantity of blood discharged in one case was half a gallon, in the other less than half a pint. In both cases the blood came away while the patients were at stool, in a coagulated condition. Connected with these discharges, in both cases, there were paleness, a sudden feeling of weakness, and almost entire subsidence of the febrile orgasm. Although hemorrhage from the bowels has been, by some modern writers, considered as a dangerous symptom, and as one for which remedies should be immediately applied, nevertheless we regarded it, in the two cases in which it occurred, as a critical evacuation, demanding no treatment whatever except the maintenance of a quiet condition of the bowels. Viewed in this light the amendment which followed its occurrence in both cases is easily understood. But crisis, by hemorrhage from the mucous membrane of the alimentary canal is as likely to take place, if we might judge, as from the mucous membrane in any other part of the body. Glass, on the "*Method of curing fevers by Hippocrates*," says, when there is "an heaviness, tension, heat, pain, and pulsation about the loins, the blood will rather burst out from the lower parts, and pass by the same way as the natural discharges." This author wrote his work a century or more since, but even in that age, hemorrhage from the bowels in typhus fever was looked upon as a critical evacuation. The hemorrhage lasted with intervals in one of the cases to which I have referred for sixteen hours; the other discharged blood but once. They were both treated with opium until the bowels were secured in a passive condition, which was maintained for several days, after which a laxative was administered, and they commenced convalescing.

Two cases were complicated with pneumonia. In one of these the inflammatory action was developed first in the pleura-costalis, and proceeded on, until the entire portion of both lungs was more or less involved. The patient, previous to the supervention of this inflammation, had become much emaciated from twenty or more days' illness; and perhaps, also, from the adoption of a vigorous course of purging with calomel and ipecac., designed to bring the fever to a termination. The pneumonia was treated with fomentations, cataplasms, blisters, almost to the exclusion of anything like internal remedies, except opiates and mucilaginous drinks. After the patient had recovered from this general affection of the chest, he was seized with a local inflammation in one of the intercostal spaces, so small in diameter that it could have been covered with a dollar. For this an epispastic was also prescribed, and the symptoms subsided. By these complications the illness of this patient was protracted for eight weeks. Pneumonia, so much to be dreaded in typhus, owes its origin not less frequently to the predisposing influence of the complaint, than to the agency of a strictly evacuant plan of treatment. Whatever debilitates or wastes the energy of the system in most of our continued fevers, favors the occurrence of inflammatory complications; because inflammations are almost as readily engrafted on a system very much debilitated, as on one of an opposite diathesis. Hence I consider the purging plan of treatment adopted in this case as having a very close relation to the trouble which followed. The other case, embarrassed by pneumonia, was that of a young man of strong vigorous constitution, full habit of body, and inclined to plethora. His fever from its commencement was rather inflammatory, and marked by a pretty decisive tendency to cerebral congestion. By the use of vigorous means the integrity of the brain was preserved, but as was subsequently discovered at the expense of the chest. Pneumonia was developed during the second stage of the fever, and although it was characterized by some degree of violence, it readily gave way under the use of purgatives and counter-irritation. This patient, if bleeding be available at all in the pneumonia of typhus, might have been bled. The pulse was frequent and tolerably hard. The disease, however, being in its second stage, I chose to sacrafice the temporary and perhaps permanent advantage of blood-letting, in order to economise the resources of the system, and obey the impulses of a firm conviction, that the 'loss and gain' of blood-letting under such circumstances are pretty well balanced.

Of congestion of the brain verging very close on inflammation, there was one case. It occurred to a female in the third stage of the disease. The stupor was so profound that the patient, for three weeks, was unconscious of everything that passed. This disturbance of the nervous influence continuing so long, the adynamia induced thereby produced a tendency in the system to gangrene; and large pieces of the integuments and muscles on several parts of the body dropped out, leaving behind phagadenic ulcers. Depletions and revulsions to the head, neck, and spine, before I saw the case, had all been, in their turn, unsuccessfully applied. To give camphor a fair trial in cerebral congestion, where the opinions concerning its efficacy are so various, I considered this a proper case. It was given in three or four grain doses every four hours, with the proper intervals; and occasionally combined with an aperient portion of calomel sufficient to keep the bowels open. Under this course for the first few days but little, if any, amendment was observed; but at the expiration of a week the efficacy of the medicine was quite palpable; and under its use with such nourishment as the digestive organs could bear, the stupor and delirium were entirely removed.

In thirteen cases diarrhœa prevailed with more or less severity. I do not mean by diarrhœa, that looseness of bowels so often attendant on typhus, and which certainly in many instances exercises a salutary influence, but I mean diarrhœa, characterized by dark watery discharges, occasionally green, in which is sometimes contained particles of the mucous covering of the bowels. Invariably the occurrence of these discharges, whether caused by medicine, or the result of disease, was attended with an aggravation of the disorder, and a rapid prostration of all the energies of the system. The pulse under its influence became frequent and feeble, the tongue red and dry, and the thirst very much increased. In a few of the cases it was accompanied with pain, in all by tenderness in the epigastrium and some degree of meteorism. Some of the cases in which this form of diarrhœa occurred had a lax state of the bowels from the beginning; it occurred to others during the second stage of the malady while under the use of the ordinary daily prescriptions, and in one or two cases it seemed to have been the result of drastic purgatives. Successfully all these cases were treated with opium and hydrargyrum cum creta. Three or four grains of the latter to one of the former was the most common prescription. In some cases however the proportion of opium was much greater, amounting to as much as three grains. This prescription was continued until the bowels yielded to its influence. In conjunction with these means I used pretty freely an infusion of slippery elm (ulmus Americana) or gum water, or flaxseed tea, as either seemed to be indicated. To the opium, however, I attributed most of the benefit derived. Nor was I prepared for confidence in the drug by previous use. Many of our elementary writers speak in doubtful terms of the efficacy of opium in cases where cerebral disturbance is at all to be dreaded, as is usually the case in typhus. Having previously had but little experience under such circumstances, I had recourse to stimulants and astringents at first, but failing with these, I concluded to give opium a fair trial; and am compelled to say that the fears I had entertained, that it would increase cerebral congestion were not realized. Every case in which it was used had more or less stupor before the medicine was given, but this so far as could be observed was not at all increased under the largest doses administered. Schoenlein, a very sensible writer on typhus, says in regard to this subject,—'the truth is we don't yet know a specific against this pernicious diarrhœa.' He however adds, 'Nux vomica with alum and gum arabic, is perhaps, after all, the best remedy.' Of the value of this I know but little. It, however, as well as the muriate of iron, brought forward recently by Autenreith, deserves to be fully tested.

Stupor more or less profound was present in every case. So entirely general in some form or other was this symptom, that we believe with Armstrong and later writers, that the membranous envelopes of the central parts of the nervous system are affected with hyperemia in every case of typhus. Nothing short of this will be adequate to explain the invariable disturbance of the nervous influence. Usually the stupor was but slight during the first stage, but after this it gradually increased until the decline of the disease, and was apparently carried off in several cases by the occurrence of a critical evacuation. In cases of this kind it was observed that the intellect recovered its usual energy much quicker than when the disease ran its course without being characterized by a well marked crisis. Nine cases had delirium combined with the stupor. For the relief of these symptoms epispastics and sinapisms were used over the spinal axis, and it appeared that the latter were most efficacious. Indeed it seems to me, that sinapisms, or any thing else calculated to make a stimulant impression, does good, not so much by their revulsive action, as they do by exciting the capillary system of the nervous investments to unload itself, so that the nerves can resume their normal action. This view furnishes an explanation of the superior efficacy of friction, stimulating liniments, and sinapisms over the nervous centres, in cases of muddiness of the intellect and many of the derangements connected with a congested condition of the nerves of organic life. Moreover, it furnishes a rational hypothesis upon which to account for the amendment which sometimes takes place in congestive diseases, by the use of internal stimulants, wine, camphor, etc.

Two cases were complicated with retention of urine. This troublesome symptom occurred to both after they had passed into the second stage of the disease, and had become very much debilitated. One had *previously* a hemorrhage from the bowels; in the other a hemorrhage of similar character *followed* the retention of urine. Caused as these cases evidently were by a want of muscular power in the bladder, they admitted of no relief except by the catheter. Daily both cases were relieved by this instrument, until the energies of the system by convalescence were competent to afford the necessary aid. While these cases were the only ones complicated by retention of urine, a majority of all the rest had more or less distress in micturition. This in some consisted in a lack of power to contract in the muscles of the abdomen and bladder, a smarting sensation in the urinary passages when voiding urine, and a dragging pain along the course of the uterus. These symptoms were referred to the debilitating effects of the disease, and the acrid character of the urine; and were treated with fomentations, flaxseed tea, and infusion of slippery elm. In several cases of this kind I used balsam copaiba, but with no marked benefit.

*Meteorismus* took place only in those cases where the virus had evidently made its deepest impression, causing the disease to be protracted. It was seldom noticed in the first stage. But after this it increased until the period of declension. To this affection, as well as a tenderness in the epigastrium, which was present in nearly every case, no treatment was specially addressed. Symptomatic, as both should be regarded, of the abdominal disturbance so generally present in typhus, the only way in which they are susceptible of relief is by a remedy of the causes upon which they depend. The secretions of gas, which take place from the mucous surface of the intestines, (tympanitis intestinalis) doubtless, when there is no obstruction in the bowels, are incapable of maintaining the regular distention of the walls of the abdomen, so conspicuous, in many instances, throughout the whole course of the disease. To the secretions therefore of the peritoneum, (tympanitis abdominalis) this condition can most reasonably be attributed. Confined closely in a shut sac, the peritoneum, it is well calculated to keep up a regular distention, and impart to the walls of the abdomen a degree of elasticity. The tenderness in the epigastrium I have often treated with counter-irritation, but the control which such means has over it, has in my hands been slight. Lately, therefore, I have regarded it as being a part of the disease itself, amenable only to general remedies; and although it was present to a greater or less extent in every case, none, so far as was observed, suffered for the want of topical applications.

It has been noticed by Schoenlein, and perhaps others, that typhus is more liable to complications when it is of spontaneous origin, than when it originates from obvious sources of contagion. This, so far as my observations have extended, is true. The first one of a family attacked is apt also to have the severe form of the complaint, while among the balance the symptoms are generally more mild. I attended seven cases in one family, all adults. In the first case the disease was most protracted, and became very much complicated, while in the remaining six it ran its course unembarrassed by any obvious inflammation. These cases occurred during the winter season, a time as we might suppose, unfavorable to the influence of contagion. But although they were in a tolerably spacious building, the exhalations arising from the body, in spite of ventilation, and the efforts to keep the skin clean, became very fetid; and hence some of the visitors contracted the disorder. None, however, had it with that degree of malignity experienced by the first. In some other instances also, where the disease appeared to originate spontaneously, the individuals first attacked suffered the most from inflammatory complications. This is very different from what might have been naturally supposed. Certainly the virus, emanating from the surface of a sick body, has the best possible chance of becoming concentrated where a number of persons are confined together in the same room. Now this being the case, persons contracting the disorder in an atmosphere of this kind ought to have it in its worst possible form. With such it should be more complicated and protracted. So far from this being the case, its opposite will more likely be found to be true. Facts of this kind must exercise some influence on our views of the etiology of typhus. More of this however in another place.

*Causes, remote and proximate.*—Long an object of the deepest interest and the most careful research, the cause of typhus fever, remote and proximate, still continues to be a fruitful theme of speculation. Alluded to from the earliest periods of medical record, the malady however has never been described as having an identity of its own until about the beginning of the eighteenth century. But before that time, as well as since, it was considered as originating under circumstances in many respects peculiar to itself.

It will be found by consulting the records of the profession on the remote cause of that group of symptoms to which, in modern times, the name typhus has been applied, that the older physicians gave about as much scope to their powers of conjecture as those of modern times; and perhaps with an equal degree of plausibility. The mystic or rather magic agent has been considered to be, a pestilential constitution of the air, the same in common with other '*continual*' fevers, (Hippocrates), the putrid effluvia of lakes and marshes, (Galen), 'foreign matter' or contagion operating upon a system poisoned with *lues venerea*, (Van Helmont), a *putrefying agent* which finds its way into the '*humors*' contained in the veins and arteries (Riverius), cold, changeable weather (Sanctorius), an epidemic constitution of the atmosphere (Sydenham), contagion (Huxham), putrid effluvia from dead animal or vegetable matter (Pringle, Cullen, Darwin, Rush, Brown), vicissitudes of temperature (Boerhaave), *malaria*, the same in common with what produces intermittents and remittents (Armstrong), an abnormal constitution of the atmosphere, in which there is a diminished quantity of oxygen associated with effluvia from animal or vegetable putrefaction (Schoenlein).

These are only a few of the conjectures which have been put forth at different eras in the profession in regard to the *remote* causes of typhus fever, jail fever, camp fever, hospital fever, etc. We call them conjectures, because all attempts to prove them true have failed. Lately, however, this subject has been approached with a degree of philosophical ingenuity by Liebig, which, like his effort on physiology, will perhaps lead to more correct knowledge concerning contagions.

He adopts the law of dynamics, proposed by La Place and Berthollet to explain the manner in which vitiated secretions act on the body in the production of fever. That law says: "*A molecule set in motion by any power, can impart its own motion to another molecule, with which it may be in contact.*" Hence says Liebig, "When putrefying muscle or pus is placed upon a fresh wound, it occasions disease and death. It is obvious that these substances communicate their own state of putrefaction to the sound blood from which they were produced, exactly in the same manner as gluten, in a state of decay or putrefaction, causes a similar transformation in a solution of sugar." "Poisons of this kind," he adds, "are even generated by the body itself in particular diseases. In smallpox, plague, and syphilis, substances of a peculiar nature are formed from the constituents of the blood. These matters are capable of inducing in the blood of a healthy individual a decomposition similar to that of which they themselves are the subjects; in other words they produce the same disease."

This purports only to show how contagious matter is reproduced in the system, and while we regard it as the most scientific rationale that has yet been proposed, we regret that the learned author was not able to give us something equally rational on the *origin* of contagion. Relating to this subject all he says is that "several kinds of contagion are propagated through the air."

Taking the law of dynamics proposed by La Place to explain the manner in which the more contagious diseases are propagated, we can see no impropriety in applying it to many other diseases of a virulent character, where the secretions and excretions of the system become vitiated and decomposed. Indeed almost any disease of a virulent character, according to this view, may elaborate its own peculiar virus, and be propagated like an epidemic. We have had some praiseworthy efforts in our own country to prove that yellow fever, the *Western* plague, is invariably imported into our Southern and Eastern cities, that it is a Southern disease, incapable of originating in the most southern latitudes of the United States; and that it never makes its appearance as an epidemic without the virus having first been imported. Of the correctness of these opinions the profession are not at all satisfied. More observation will be necessary to settle these difficult and important points in the history of yellow fever. Generally, however, there is a belief prevalent that the disease after it has made its appearance, may elaborate its own peculiar virus and thus propagate itself. Many practitioners on the continent of Europe believe this to be the case, and also, more or less, in regard to all fevers, even puerperal.

Previously it has been remarked that the spontaneous cases of typhus, or those originating from a *constitution of the air* were more violent than those in which the disease was seemingly contracted from persons already laboring under its influence; and it has been observed by some writers in Great Britain and Germany, that typhus is more violent in the country than in the city. As it regards this violence of the spontaneous cases, it will perhaps find its best solution by considering the cause which gives rise to them, as being different from that by which the disorder spreads through a family, camp, jail, or hospital. The former, by pretty general consent, are due to an epidemic constitution of the atmosphere, the latter probably depend upon the infectious matter generated within the body, which, while it is capable of producing a disease having all the characteristics of typhus, it nevertheless produces it in a modified form. Why the disease originating in the country should be more violent than when it occurs in a city, is a problem that in the present state of our knowledge cannot be solved. We would suppose *a priori* that the effeminate habits incident to city life would beget the most favorable condition in the system for the invasion of the virus; and, as a matter of course, it should in such cases display its most malignant effects. Such, however, is not the case, and we are left with nothing better to account for the fact, than mere speculation.

Confessedly obscure now as the *remote* cause appears to be, there are nevertheless some circumstances connected with its origin and propagation that are better understood, and are capable of being regarded with some considerable degree of certainty.

1. Typhus fever, according to present experience, is indigenous, or belongs exclusively to the northern hemisphere, and only exists in the southern by being carried there.

2. As a general rule, typhus prefers low countries. This peculiarity has been observed as being pretty constant, since Francastori first described *petechial* typhus in 1805; yet it occasionally ascends to immense heights; for example the petechial typhus of 1814–'15 went up to the highest Alps. But in such situations the cases were more rare and attended with less severity.

3. A moist condition of the atmosphere, low range of the thermometer, rapid changes in the temperature of the atmosphere, yet within certain limits, seem to be favorable for the development of the contagion.

4. Every thing calculated directly or indirectly to exercise a paralysing impression upon the nervous system, such as the depressing passions, strong mental exertions, exhaustions of the muscular system, and exposure of the skin to wet and cold, prepares the system for an attack of the disease.

5. Personal filth, a want of wholesome nourishment, bad water, bad clothing, and everything calculated to favor putridity, as the crowding together of a number of persons in the filthy apartments of a camp, ship, jail, hospital, or in the sordid hovels of the poor, predispose the system to the influence of the virus.

6. The contagion finds the greatest susceptibility to its influence in the middle period of life, the period of the highest development of the nervous system. Hildebrand says that young children who in the general are so susceptible of contagious diseases, are seldom affected with typhus. Equally seldom is the disease met with in the aged or withered individual.

7. In a pure, free air, the typhus contagion only extends from the person affected, to the distance of a few feet, three or four; hence if proper precautions are observed in cleanliness and ventilation, the spread of the disease may always be prevented.

Imperfect as this enumeration may seem to be, it nevertheless comprises most of what we know in regard to the remote cause. Concerning the proximate, although much more important, the views of the profession are still somewhat unsettled. And though it may not minister much to professional pride to retrospect the chimerical notions of many of the ancients, yet regarded as mere matters of history, they may be profitable in moderating the dogmatical opinions, if they have any, of the more learned pathologists of the present day.

To begin with Hippocrates—he considered the proximate cause to be morbific matter in the circulating fluids; Galen, friction between the solids and the fluids by morbific matter in the blood; Bellini, lentor or viscidity in the blood; Van Helmont, excess of fermentation among the solids and fluids of which the body is composed, or 'Archeus' defiled by a foreign idea; Riverius, putrid humors in the blood; Willis, a collision of nitre with sulphur, in the blood; Sydenham, fermentation in the blood, similar to that of the vinous in vegetables; Huxham, lentor and vapidity of the nervous juices, together with poor weak blood; Sanctorius, perspirable matter retained in the blood during the cold season of the year; Ball and Boerhaave, acrimony in the blood; Brookes, putrid fermentation in the blood; Baglivi, a venomous ferment; Pringle, Marcus and Clutterbuck, an inflammation of the brain; Hoffman and Cullen, a spasm or constriction of the capillary arteries on the surface of the body, attended with a diminished energy of the brain; Darwin, a disorder of the stomach; Brown and Rush, debility; Wilson, a change in the laws of excitability producing atony; Gregory, a defect in the functions of the brain, and spasm of the extreme vessels in every part of the system; Armstrong, a simultaneous congestion of the brain, lungs, and alimentary canal; Hildebrand, inflammation of all the mucous membranes; Broussais, gastro-enteritis with adynamia; Roederer, Wagler, Prost, M.M. Petit, Serres, Louis, Cruveilhier, Bright, and Carswell, a diminished cohesion of the solids and fluids which compose the body, and a morbid alteration of the intestinal, chiefly the Peyerian glands.

These are a few of the many opinions maintained at different periods in the history of the profession. With many, and among whom may be ranked the father of medicine, all fevers were considered as having their origin in a common cause, varied however, in form or type, by the state of the atmosphere, locality, idiosyncrasy, age, etc. But among all there is a vein of unanimity, that in the group of symptoms to which the name typhus, in modern times, has been applied, there is a putrid or adynamic condition of the system. With the older pathologists, and even down to the times of Hoffman and Cullen, this putridity was almost exclusively confined to the blood. Since their day, and perhaps from their influence, the solids have come in for a share of the credit due to abnormal action, not only in typhus, but all other kinds of fever. Now the profession stand divided, with the preponderance, probably, in favor of the solids being the primary seat of the diseased action. The truth is, without doubt, as it has been laid down by many eminent pathologists of the present age, between the two extremes. Why the fluids, that constitute four-fifths of the body, should escape, and remain uncontaminated during the course of a general disease, where the organic changes in some instances amount almost to a metamorphosis of every thing relating to the system, is so astonishing that independent of demonstration, it cannot be believed. If we can trust to recent research, the blood is identical in composition with that of flesh, (Liebig) and as a matter of consequence, liable to the same kind of lesions; or may we not, owing to the constituents of which it is composed, and their existence in the fluid state, say with propriety that it is more liable? Waving this subject for the present, we may remark that the literature of the profession, by general consent, makes the pathology of typhus to consist in an anatomical condition involving generally the lungs and intestinal canal. Constantly there is, as Armstrong has observed, more or less congestion of the mucous lining of the air passages, amounting, in many instances, to bronchitis or pneumonia. In the intestinal canal there seems nevertheless to be the most important and most constant alterations. So generally has this been observed that in his last edition of the Clinique Medicale, Andral ranges fevers under the head of abdominal diseases. The lesions are commonly found in that portion of the ileum situated near the cæcum, occasionally extending to the cæcum and colon, and consists in inflammation and ulceration of Peyer's glands. From the fact that this structural change is so invariably present, many have regarded it, rather as a part of the disease, an almost essential part, bearing the same relation to the entire disease which the rash and sore throat do to scarlatina. Whether softening of the parenchymatous substance of the different organs, the brain, heart, liver, spleen, the kidneys, the softening of the mucous membranes, the rupture of the textures of the skin, the mucous, and serous membranes, giving rise to petechiæ, vibices and effusions of blood, and the diminished cohesion of the blood itself, are parts of the disease of an essential character, or anatomical conditions with which it is occasionally complicated, remains to some extent unsettled. If any lesion besides that of the chest and intestinal canal possesses a share in the pathology of typhus, we think it due to the nervous system. We meet with so much in a case of typhus, referable to a functional or structural disorder of the brain, such as great prostration, the different varieties of delirium, flaccitation, *subsultus tendinum*, tremors, and paralysis of the sphincters, that, with the German physicians generally, we might conclude that the nervous centres are also affected, in a manner sufficiently constant to make it a grave question, whether or not it makes up a link in the chain of morbid actions which take place in this malady.

*Are typhus and typhoid fevers identical?* This question, which may be regarded as dividing the pathologists of France and Britain; and to some extent exerting a similar influence among the practitioners of the United States, is one which perhaps, will remain unsettled during the present generation. We have no doubt, but that the *low* fever of Hippocrates and Galen, the *tumulus pestis* of Van Helmont, the *putrid malignant* of Riverius, the *pestilential* of Sydenham, the *malignant* of Bellini, the *putrid* of Sanctorius, the *continual putrid* of Boerhaave, the *malignant*, *goal*, *hospital* or *camp* of Pringle, the *putrid malignant* of Huxham, the *petechial* of Francastorius, the *putrid* or *spotted* of Brookes, the *mesenteric* of Baglivi, and the *typhus* or *nervous* of Cullen, Thomas, Armstrong, and modern writers generally, are identical in most of the phenomena presented, and might safely be regarded as one and the same disease, varied only by locality, different atmospheric constitutions, habits of living, etc. Concerning the prostrated putrid condition of the system, the diarrhœa, and other phenomena, now regarded as being essential to typhus fever, there is a striking agreement between the most antiquated accounts of the prevalence of this malady, and those given to us by modern writers.

But that the typhus of Armstrong and the typhoid of Louis are the same disease, is perhaps not quite so certain. Our opinions nevertheless incline to the position that they are, and the reasons for these opinions will now be submitted.

In doing this we will first give the points, in which there seems, between the two diseases, to be a resemblance:

1. Both may have an eruption, (petechiæ or sudamina) and both may prevail without any.
2. In both the prostration is alike.
3. In both the anatomical lesions of the digestive and nervous systems are much the same.
4. The bronchial and pulmonary disturbances, occasionally present in both diseases, are alike.
5. The pulse is alike in both; and the weakness of it in both diseases, without doubt, depends on softening of the left ventricle of the heart.
6. In both there is a tendency to bed-sores.
7. Both may prevail with severe anatomical lesions; and both may prevail without any at all.

On this subject Andral, in his Clinique Medicale, says that patients have perished marked with all the symptoms of *typhoid* fever, and yet there was neither exanthema, ulceratons, nor appreciable alterations of any kind in the digestive canal, the place where, of all others, alterations or changes of texture are most likely to be found.

The points in which the two diseases are said to differ are:

1. In typhus the nervous centres are principally implicated; in typhoid, the intestines.
2. Genuine typhus is reported to be highly contagious, typhoid is said to be not contagious.

With regard to the first of these reputed points of difference there is a great diversity of opinion. The nervous system evidently suffers more or less in every case of genuine typhus, and may safely be regarded as one of the symptoms which are pretty constant. Indeed the disturbance here is occasionally so great as to produce softening in the brain.

But is the brain free from disease in typhoid fever? If it is, to what are the diagnostic symptoms of this fever, as given by Andral referable? such as headache, diarrhœa, *stupor*, *delirium*, *somnolency*, etc. Demonstratively to us, the stupor, headache, somnolency, and delirium, point to lesions of the brain. And while such symptoms continue to be given as pathognomonic by the most learned in the profession, we cannot see how they can be otherwise interpreted. There is then in the disturbance of the nervous system a similarity instead of a difference between the two diseases.

Of the intestinal lesions so predominent in typhoid, the same my be remarked. Writers of the greatest antiquity speak of diarrhœa as a complication of typhus; and Van Helmont says, that the seat of malignant fevers is "in the first shops," the intestines. Dr. N. Smith, of our own country, says, that he never knew a case of typhus to terminate fatally without diarrhœa. Dr. Tweedie, however, in his Clinical Illustrations of Fever, and Armstrong in his work on Typhus Fever, give functional and structural changes of the intestinal tube as being among the prevailing lesions.—To these authors we might also add the names of Hall, Bright, Carswell, and others, perhaps not less distinguished, but we submit the matter, with what we have advanced, for candid consideration, believing that those who will take the trouble to analyse the literature of the subject, will come to the conclusion, that so far as intestinal lesions are concerned, there is no difference yet made out between the two diseases.

As it regards the other point in which there is said to be a difference, viz: that typhus is contagious and typhoid not, we think the reasoning relied upon to prove it, wants that conclusiveness adequate to produce anything like general conviction.

Invariably all who have any experience in typhus, award to it the power to propagate itself by contagion. But it also originates spontaneously, apparently from what was called in former times an epidemic constitution of the atmosphere.

Well, how will these traits compare with the disease called typhoid? In the preceding part of this paper, we showed that if we could rely upon the researches of such men as Liebig, we were compelled to admit that any disease capable of producing a decomposition of the secretions and excretions of the body, or of corrupting the fluids, might elaborate a peculiar virus by which it might propagate itself, and prevail according to all the known laws of contagion. Without, however, relying upon this general principle, we are enabled to state upon good authority, that the disease which Bretonneau calls *dothinenteritis*, which Andral calls *follicular enteritis*, which Roederer and Wagler call the *mucous fever*, which Broussais calls the *adynamic gastro-enteritis*, and which Louis, Chomel, and others call the *typhoid fever* or *affection*, is acknowledged by Lombard, Bretonneau, Gendrin, Chomel, Louis, and others, to spread and propagate itself by contagion.

Here then are all the reputed points of difference melted down by nothing more nor less than the simple process of analysis. Our reasons, *in limine*, may now be summed up for regarding the two diseases as identical: 1, because they are alike in *history;* 2, in *symptoms*, and 3, in *pathological anatomy.*

We have adverted to this subject for the purpose of eliciting inquiry concerning the character of what is called in the Western States '*Winter* fever,' '*Winter typhus*,' '*low, continued*, or remittent fever,' '*typhoid fever*,' *&c.*, as in our opinion there is reason to suspect a closer affinity between these diseases and genuine typhus than has generally been supposed. By examining the reports of them, as they have prevailed in Ohio, Indiana, Illinois, and other Western, and also in the South-western States generally, we find that the general symptoms correspond with what in typhus we regard as pathognomonic. Dr. Carrol, now of Cincinnati, in the *Western Journal*, described a form of this fever which prevailed in Belmont county, Ohio, as early as 1814. Various indeed have been the sources from which we have received information of its prevalence at different times. Within the bounds of my own practice I have noticed more or less of it for the last six years, and can now hear of its prevalence in other parts of the State, called by various names, and so far as its pathology is concerned being very differently regarded. Confident, as we ourselves feel, that what has prevailed under all these various names, is nothing more than the old fashioned typhus wearing a new livery according to the localities, idiosyncrasies, habits, &c., to which our population is exposed, we nevertheless feel that such a generalization ought to be made the subject of the severest criticism; and if this be grounded upon an observation of one of these epidemic fevers, or all of them together, we will cheerfully abide the issue.

*Course and Termination.*—Undecided to a very great extent have the profession been on this subject, since the days of Hippocrates and Galen. Many have regarded the course of typhus as amenable altogether to the laws governing inflammation, for to this in some form or other, they have imagined the pathology to be due. While others have regarded it as being under the influence of a law of self-limitation, and pretty much confined in its course to the seven days period. The seeming difference in these views, we think, by properly considering the nature of the disease, and the complications to which it is liable, is capable of being reconciled. The critical days, of which authors speak, we are willing to admit, have their foundation in a correct observance of the disease. According to Hippocrates and Galen, the greatest number of fevers terminate favorably on the 7th day, and many on the 14th. Next to these in order of efficiency, are the 9th, 11th, 20th or 21st, 17th, 5th, 4th, 3d, 18th, 27th, and 28th. In the correctness of these periods, that part of the profession, believing at all in critical days, have generally concurred. The disease so far as our own observations are concerned, has observed the seven days period, terminating at the 7th, 14th, 21st, or 28th day. Now we conclude that the disease observes these periods in its course, when, as was remarked by an old writer of some eminence, 'it seems to have its seat everywhere and nowhere.' That it does run through its course in this way very frequently, we have testimony the most abundant. And the notes now in my own possession, taken from its prevalence among us within the past year, show a number of cases in which, from the commencement to the close, the seven days period was strictly observed.

Frequently too we have observed it pay no attention at all to periods of any kind. In cases of this kind, in the literal acceptation of the term, it prevails as a '*continual*' disease; and seems to leave the system with as much tardiness as does a chronic inflammation. What now can be assigned as the cause of this difference in termination? The true explanation of it will be found in the fact, that in addition to the proper pathology of the disease, there occasionally supervenes an inflammation, that subverts the governing law of self-limitation, and protracts the duration of the malady to a time commensurate with its own intensity and extent. In this way we account for the diversity of opinion extant on this subject; and believe that the disease may prevail according to the seven days period, or without seeming in any respect to be governed by such a law.

*Can the disease be cured?*—This question, if designed to inquire whether the disease is capable of being shortened in its course, or cured at any stage without reference to the laws by which it is governed, is one, that has been warmly contested. When the malady prevails in its simple form uncomplicated with any obvious visceral inflammation, it seems to be prone to run its course regardless of all the remedies that may be administered. From this circumstance many hold the doctrine that typhus has always to run through a certain cycle of changes, from which it ought not to be moved; and hence the plan of treatment, most successful with them, savors strongly of that of the *expectant*. With some it consists literally in doing nothing. Others make a show of treatment by the use of mere temporary palliatives and trust the final issue to the occurrence of critical evacuations, in the urine, through the skin, or by furuncle, abscess, parotitis, or hemorrhage. Among this class of practitioners in typhus, can be found the names of some of the most distinguished men in the profession. Bold and vigorous as was the practice of the celebrated Rush, he, both by precept and example, inculcated, in all simple cases, the palliative plan of treatment. In his judgment the business of the physician consisted in standing as a sentinel, not to expel the enemy, not to enter a crusade against his right of possession, but to guard as much as possible the integrity of the most important parts of the invaded dominion. This plan, although often laid aside as being antiquated, and we might almost say as being criminal, for it has been called, since the time of Stahl, '*a meditation on death*,' has nevertheless, by the success which has attended it, secured no small share of confidence in modern times. Even as late as his edition of Stokes' Lectures, Dr. Bell says, "But with whatever industry and judgment we may follow up the practice, the heads of which I have just sketched, typhus will still persist in its course, after a while to take a salutary turn towards recovery and health, or to assume a more aggravated and complicated character." Different from this plan however only in a few minor points, is that pursued in Paris, the Athens of medical literature, at the present day. True, indeed, they bleed in their *typhoid affection*, in most cases, to a sparing extent. But remove this from their practice, and all the balance of their means may safely be regarded as perfectly effeminate. Distrust in the efficacy of other therapeutic agents seems to stick out of every clinical report received. This must proceed from a consciousness that the disease has a cycle of changes through which it will invariably pass; and that medicines thrust upon the system with the intention of breaking this up, are more apt to produce complication than cure. Any other explanation of the French practice, we look upon as incompatible with that boldness and eminence in other departments, for which they are so justly distinguished.

Taking it now for granted that simple typhus, like the exanthemata, has a certain course through which it will run, irrespective of any of the known agents of the materia medica, we proceed in the next place to notice what are the most available means when it is simple, and also when it becomes complicated. And here it may be remarked that in looking over the materia medica we can scarcely find an article that in typhus has not been more or less used. The limits of our remarks however will prevent us from considering, only in a brief manner, the more important, such as are calculated, from their acknowledged superior powers, to fulfil in the best manner the more important indications that in the course of the disease may be presented.

Standing foremost, if not first, among these may be ranked the preparations of mercury. Used too as this article has been by every member of the profession, from the merest tyro to the most exalted genius, there is nevertheless a humiliating deficiency in our knowledge of its utility in fevers in general, and typhus in particular, that seems almost unpardonable. Immense doses, as well to test its efficacy, as from a desire to ascertain its physiological effects, have been given during the late prevalence of cholera. But these doses, contrary to what had been the generally received opinion, seemed to act as a *sedative*, by the manner in which the vomiting and purging were allayed. Charles Bennet, house-surgeon of the Cholera Hospital at Bethnal Green, however, reports a case in which 53 drachms of calomel were administered within forty-two hours without the least sensible effect. Other cases are related, in which from large doses, the most distressing consequences ensued. Wilmer, Gmelin, and others, report cases where large doses produced not only irritation, but other effects, in many instances fatal in their character. The same diversity of opinions on the *physiological* effects of small doses obtains. Many regarding small doses as decidedly irritant, while others regard them as most free from all the objections that have been urged. "These statements," says Pereira in his late work on Materia Medica and Therapeutics, "appear to be almost inconsistent, and yet they are fair deductions from the experience of numerous intelligent practitioners. We must therefore," he continues "endeavor to accumulate more facts, in order to illustrate the effects of calomel, and for the present confess we have very imperfect information respecting the nature of its action."

Such are the conclusions of Pereira, the latest and perhaps most comprehensive writer on materia medica.

Of the utility of exciting the mercurial disease, in order to cure fever of any kind, our opinions have heretofore been given to the profession. All that we have to add now therefore, to those already expressed, would only go to confirm the position that such practice, as it has been witnessed by us in typhus fever, is, if we judge by the mortality attending it, extremely doubtful. Instituted at the onset of the malady before the disease has passed the period of incubation, we can readily suppose it capable of breaking up the train of morbid action and assuming for a time the control of the entire organism. But when typhus becomes seated, displaying its ravages upon the viscera of the great splanchnic cavities, like the well-forted aristocrat, it rules and governs to the exclusion of every thing else. We have made attempts to rout it, time and again; and, after wasting no little of the energies of the system, were at last foiled.

For the *hydrargyrum cum creta*, among all the preparations of mercury, we have a decided preference. Our usual mode of preparing it differs, likely, in some respects from the officinal. We have been in the habit of taking the blue mass and a sufficient quantity of prepared chalk and rubbing them together in a mortar until they make an impalpable powder, which, by varying the quantity of the blue mass may be made of any strength desired. This preparation we have used in the treatment of typhus for some years, and our confidence in its efficacy has never been diminished. We use it in all the stages of the disease, as an aperient, as well as to allay, in combination with opium, the distressing diarrhœa usually attendant. In cerebral congestion we combine camphor, and in the mucous engorgements and irritation of the air passages which frequently supervene we combine it with *pulvis Doveri.*

Prominently standing forth do we also find the preparation of *antimony*, tartar emetic. Since the time of Rasori the attention of the profession has been frequently called to the usefulness of this drug, as well in typhus, as in other varieties of fever. With the physiological effects of the medicine on the surface of the skin all are familiar. It is a powerful local irritant, and when applied to the epidermis it causes an eruption of painful pustules, resembling those of variola, or ecthyma. This being its effect when applied to the epidermis, the strong resemblance which is now universally admitted to exist between the membrane on the exterior of the body and the mucous membranes generally, even in the absence of other facts, would teach us something concerning the physiological effects of tartar emetic, when applied to the mucous membranes. But we are not destitute of some evidence on this point. We have witnessed tartar emetic pustules on the inside of the mouth, after having used a solution of the medicine for several days in succession. Further, Pereira states, "that a similar pustular eruption has been met with in the mouth, œsophagus, and small intestines from the internal use of tartar emetic, and white apthous spots have been observed on the velum and tonsils."

Having these facts before us we are prepared to estimate the value of tartar emetic in the treatment of typhus fever, which of course must vary, more or less, according to the stage to which it is applied, or the indication which it is intended to fulfil.

In the commencement of the disease, the period of incubation, the medicine, given in doses sufficiently large to produce emesis, will in many cases break up the train of morbid symptoms, and make such an impression upon the system as will entirely subvert all diseased action. To this stage of the disease the usefulnsss of the medicine is mostly confined.

We are aware, however, that Rasori and Laennec, in view of what they supposed were its '*contra-stimulant*' properties, gave it in scruple and drachm doses. But such practice, while it may be sustained above suspicion by the authors to whom we have alluded, in the treatment of violent inflammatory disease, can certainly secure but little confidence in the management of typhus, a disease in which the physiological effects of the medicine could not fail to be productive of serious mischief, by coming in contact with the mucous surfaces of the bowels, already too much predisposed to ulceration.

As a febrifuge proper, to allay or mitigate the febrile orgasm, particularly when the excitement of the heart and arteries is great, the medicine is entitled to the highest consideration. Rasori, the founder of the new Italian doctrine of '*contra-stimulus*,' was in the habit of administering small doses of tartar emetic during the twenty-four hours as an antipyrectic, in an epidemic petechial typhus which prevailed in Genoa. This practice soon found its way into France, England, and the U. States, and may now be regarded as sharing more or less freely of the confidence of many eminent physicians. The opinion is pretty general that it exercises a *sedative* influence over the circulation. But all attempts to explain its *modus medendi* are unsatisfactory. Scarcely two agree in all their views. Rasori thinks that it has the power of directly diminishing the inflammatory diathesis. Broussais ascribes its curative powers to its revulsive action on the gastro-intestinal mucous membrane. Fontaneilles ascribes it to alterations in the composition of the blood. Eberle thinks it due entirely to its sedative influence. McCartney regards it as a medicine diminishing the force of the circulation by the nausea which it occasions. Amidst all this diversity of opinion on the *modus medendi*, there is nevertheless much unanimity of sentiment among these writers on the efficacy of the medicine as an antiphlogistic, and to subdue the vascular excitement that takes place during the exacerbation of fever. Compelled as we are to grant that tartar emetic does exercise an anti-pyrectic influence in the stage of excitement, and that it has considerable claims to the character of a febrifuge, still we think its use, in even this condition of a case of typhus fever, to be of doubtful propriety. While it would allay the febrile orgasm, it could not fail to stir up a degree of irritation in the bowels which would countervail all the advantages obtained. This objection then, founded upon its physiological effects, lies against its continued use in fever, no matter what our views are of its *modus medendi.* We know that there is a strong similarity between the mucous membranes and the skin; we know the effects of the medicine when kept in constant application to the skin, and with these data, independent of the observations to which we have already alluded, we think the effects of the medicine on the mucous surfaces, cannot easily be mistaken.

We have another view to take of tartar emetic, and that is the one to which the attention of the profession has lately been called by Dr. Graves. To do justice to his views we will give the leading symptoms, for the relief of which he believes the medicine available. In a lecture on the spotted fever epidemic in Dublin, in 1834–'35, he says: "I wish you clearly to understand, that, after the headache and cerebral excitement which accompanied the fever, had been subdued, or had ceased; after sleep and calm had returned, and continued for many days, then a new order of things commenced; *subsultus*, watchfulness, muttering, raving, involuntary discharges, &c., all denoted great derangement of the nervous system; but still there was no proof that this derangement depended on cerebral congestion. After a few, or after many days, however, unequivocal symptoms of the latter set in; the face and eyes became suffused and flushed, the pupils manifested a tendency to become contracted, and occasionally convulsions took place, the patient became totally sleepless. When the latter and dangerous period of the fever was accompanied by the former nervous group of symptoms *alone*, they yielded to wine, musk, porter, and opiates; but when the symptoms indicating cerebral congestion were superadded, then it was that the case assumed so great and striking a similarity, so far as the nervous system was concerned, to the well known variety of *delirium tremens*, accompanied by cerebral congestion,—to that variety of *delirium tremens*, in fact, which can only be successfully treated by the judicious, but bold exhibition of tartar emetic, combined with laudanum." Here then it is affirmed, that when we have cerebral congestion added to the other, and we may truly say, dangerous symptoms attending the last stage of typhus, the remedy is tartar emetic and opium. Of the utility of this practice, to which Dr. Graves lays claims of originality, we know almost nothing. We do, however, respect the source from which it comes, and intend in future to give it the benefit of a fair trial. In the meantime we should have a different opinion of it, had the tartar emetic, uncombined with anything else, accomplished such good results. For while the removal of such grave symptoms, as wild, raving delirium, subsultus, muttering, and involuntary discharges, by an antiphlogistic, and that too, when they are associated with cerebral congestion, seems doubtful; we can, nevertheless, more readily understand how all these good results might have been brought about by opium *alone*. Some writers now claim for opiates the accomplishment of just such ends. We lately came to the knowledge, that some intelligent physicians of our own State were using opium as a kind of febrifuge, and, from their reports, with some degree of success. Administered in the last stage of typhus, it has accomplished more in my hands than any thing else that I have used. Small doses stimulate, while larger ones produce a sedative effect. Nor have I observed it to increase, or favor, in the manner mentioned by some writers, congestion of the brain. From circumstances of this kind we cannot see the propriety of attributing to tartar emetic the accomplishment of such important results, results too, which, if properly understood, may be due to the article with which it was combined. At any rate, such results are more in accordance with what might have been expected of opium, than of tartar emetic; and hence, to us, the good effects, of which Dr. Graves speaks, following this preparation of antimony, in the condition of the system to which he has refered, are of a character that leaves the subject in ambiguity.

As it regards the utility of bloodletting in this affection, there is less unanimity of sentiment than upon almost any thing which has made up a part of the treatment. While some practitioners bleed by the pint, and inculcate the propriety of the practice with a degree of enthusiasm bordering almost upon the belief that it is a specific, others there are, that withhold the lancet entirely, and look upon the abstraction of blood in any stage of the disease as being decidedly contraindicated. Now that this should be the case, after so much research into the nature of the disease, and so much experience, as it regards the most successful plan of medication, is certainly very strange. In most other disorders the profession, by general consent, have agreed, either upon the indication that this agent is calculated to fulfil, or upon the particular circumstances regulating its employment. But in typhus, the recorded and unrecorded opinions are perfectly dogmatical, both for and against it. But this is not all: these practitioners alike, when called upon to sustain their positions, forestall immediately all opposition to their views, by appealing for evidence to the success of their modes of treatment. In all this there is something wrong, and the truth, without doubt, lies to some extent between the extremes.

Among the majority of writers on typhus, there is a concurrence in the opinion that it is always characterized, more or less strongly, by a putrid diathesis of the general system. This opinion is not less prevalent among our best modern writers, than among those of antiquity. This being the case, antiphlogistic remedies in general, at the head of which stands bloodletting, are certainly of doubtful propriety—contra-indicated, we might almost affirm. The remedy then, if we have any correct conceptions of its effects, could not act otherwise than detrimentally upon such a state of the system. Applied as these remarks are intended to be, to mild and simple cases, there may nevertheless circumstances arise, during the course of the malady, where sanguineous evacutions may be more reasonably sustained.

When the disease supervenes upon a young subject with full or plethoric habits, the disturbance to which the sanguiferous system must necessarily be subjected, would create, if admissible in any situation, a necessity for depletion by the lancet. But even here, we must not overlook the fact, "that the vivifying agency of the blood must ever continue to be the most important condition in the restoration of a disturbed equilibrium, which result is always dependent on the saving of time; and the blood must, therefore, be considered and constantly kept in view, as *the ultimate and most powerful cause of a lasting vital resistance*, as well in the diseased as in the unaffected parts of the body." (Liebig). This is a recognition of the propriety of sustaining the integrity of Cullen's *vis medicatrix naturæ*, which, if ever of paramount importance, is certainly so in the treatment of a disease in which, throughout the whole course, the vital resistance of the system appears to be primarily impaired.

In that variety of typhus, denominated by Armstrong and later writers *congestive*, many practitioners favor the abstraction of blood. Different, to a very great extent, are the views of the profession on the pathological condition which the term congestive, when applied to the encephalon, is meant to express. If we are to understand by the term a loaded condition of the nervous system of the neurilema surrounding the nerves of animal and organic life, the practice would differ very essentially from that which should be adopted, were we to understand the term congestive to mean a muddy. stupid, or comatose condition of the intellect, proceeding from fullness and activity of the arterial system. For the former, stimulants, something calculated to excite the torpid capillary veins to contract and disgorge themselves, would be indicated; while for the latter nothing would be so available as local and general bloodletting. We may remark here that the Germans, and some of the French, look upon this cerebral disturbance as being of the former character, depending on venous congestion; and hence they rely almost entirely for its removal upon stimulants. In quite a number of the cases previously noticed in this paper, the intellectual functions were very much disturbed; but looking upon this as a part of the disease, little or nothing of special treatment was instituted. The issue, however, of these cases was favorable; as much so, I believe, as in those where this symptom received more attention. The only case in which the symptoms might have been considered of a very grave character was treated with camphor, and stimulating ptisans. This treatment was instituted some five or six days after the lancet and revulsives had been used unsuccessfully; and the amendment under its influence was obvious in a very short period. It might here now be observed, that so far as our own experience is concerned, we have never yet seen a case of typhus with any grade of cerebral distress, benefitted by bloodletting either local or general. From this circumstance, no less than from the adynamic character of the disorder, we look upon the symptoms, simulating arterial excitement of the brain, as being fallacious, and only capable of being removed by the judicious use of stimulants.

As hemorrhage very frequently occurs in typhus, it has been suggested that the timely and judicious employment of the lancet is calculated to prevent this occurrence. Those who insist upon this point should recollect that the hemorrhage here is nothing more than a critical evacuation; and it would be about as philosophical, we conclude, to institute measures to suppress a critical sweat, or a critical deposit in the urine, as to attempt to anticipate or stop a critical hemorrhage. Besides, those who have adopted this practice report nothing in its favor. In the only case of typhus fever out of fifty in which he bled, Dr. N. Smith says, that he had hemorrhage.

Decidedly as we design to present our views on this subject, we do not however intend to say that patients will not get well after being bled, or that in many cases they will not recover with apparent facility. We know that in many cases there is great tolerance of the loss of blood, and that in others considerations of various kinds may justify the use of the lancet. But its use in simple cases, or to make an antiphlogistic impression upon the system, or to attempt to subdue the cerebral disturbance, or anticipate a hemorrhage, we think to be of doubtful propriety. Simple cases run their course better without bloodletting, while the cerebral disturbance will be found to be amenable to very different means; and so far as hemorrhages are concerned, granting them to be prejudicial, in any stage of the malady, which, by the by, we do not, we can see no more propriety in the hemorrhage from the orifice of a lancet, than in a hemorrhage from any of the mucous membranes. If the one is prejudicial, so must be the other. Nor do we believe that the position could be at all strengthened by appealing to the fact, that in the one case the blood is abstracted at the commencement, in the other at the close of the disease; for, if the presence of the blood is necessary to keep up and maintain the vital resistance against the encroachments of the malady, it is a matter of minor consideration, so far as the welfare of the system is concerned, at what particular time this vital resistance is disturbed or removed.

Of the value of *counter-irritation* we are, from pretty extensive use, well convinced. Merely for the purpose of subduing pain and acute inflammation, we use blisters. These, however, have not proven themselves to be as available in the sub-acute inflammation with which typhus is frequently complicated, and in the removal of the cephalalgia, stupor, coma, and delirium which usually attend severe cases, as stimulating cataplasms and mustard. Applied to the spine and nape of the neck, these means often control the pain of the nervous centres, and moderate the morbid excitement of the involuntary functions of organic life. The distress of the head too we have observed to be strikingly mitigated by the application of sinapisms, and other stimulating substances, to the nape of the neck. But counter-irritation, even in the form of stimulating frictions, constantly kept up on the extremities, will, if instituted early in the disease, do much towards a favorable issue by keeping the excitement *in equilibrio*, and thus preventing congestions from taking place in the deep-seated organs of the body. Tepid vinegar and water, when the central heat predominates, and the skin is dry, husky, and constricted, will, if applied regularly, and wiped off with a coarse towel, not only favor the return of normal secretions in this organ, but by the derivative effect produced upon the surface lessen, to a very considerable extent, the danger of visceral inflammation. On this account we have not attended such a case of typhus for some time, without having this treatment faithfully observed; and we have thought always with the most decided benefit.

Concerning the availability of stimulants in this affection there are, as on bloodletting, widely different views. Some of our most intelligent practitioners use brandy and wine profusely throughout the whole course of the disease, while others place most confidence in means of an exactly opposite character. Our own views of the state of the system, and the pathology of the disease, together with what information we have obtained from the effects of remedies, induce us to favor the stimulant more, at any rate, than the contra-stimulant plan of treatment. Much, however, will depend upon the judgment of the physician in properly ascertaining the condition of the system to which they are applicable. In cases complicated with visceral inflammation of any kind they are, if not inadmissible, productive of but little good. It is true that we sometimes make an attempt to hold up the system of a debilitated patient with stimulants, while we unloose the fangs of an inflammation upon some vital organ, but it is very probable that the good and bad effects of such practice are pretty well balanced. The circulation in such cases, according to our observations, is generally increased in frequency and rendered more feeble. When this is the case, it may be taken for granted that the use of stimulants is contra-indicated. These agents, in the language of a late author, Pereira, are designed 'to vivify or strengthen,' and when they produce this effect in typhus, it is seen in the fullness and slowness of the pulse, alterations in the circulation which denote that these agencies have been rightly applied.

About the relative value of special stimuli we have different opinions. Some confide almost exclusively in ammonia, while others have derived the best effects from the different preparations of alcohol. So far as we are acquainted, there has been but little said by writers upon the conditions of the system, or the indications of the disease, which would give a pre ference to one stimulating agent over another. There is perhaps something in the temperament of patients, or in the diathesis to which they may be predisposed, which should be regarded when making selections of a stimulant suitable to their case. And again, we know that alcohol makes its principal impression on the nervous system, ammonia on the cardiaco-vascular, and the spices make theirs on the gastro-enteritic. Circumstances of this kind will by no means justify the indiscriminate use of stimulants in all conditions. Hence, when in the course of the disease a stimulant is indicated, much will depend upon making a right selection—as much perhaps, as in making the right selection of a purgative or an emetic.

A very important agent in the treatment of typhus fever is cold water. To us it seems to have considerable claims to the character of a febrifuge. Used either externally or internally it possesses more control over the temperature of the body, than any thing with which the physician is acquainted. Our practice is to allow patients to drink *ad libitum;* and also to use it freely upon the skin when the heat is great, and when this organ is contracted. With the conditions of the system by which its use is contra-indicated all are familiar; and as we have elsewhere (Western Journal) given our views on the use of cold water in remittent fever, we shall not discuss the subject further. We however would add, that we have not seen either rheumatism or pneumonia, as a consequence of the use of cold water in typhus, as contended for by Clutterbuck. (Inquiry into the Seat and Nature of Fever).

In conclusion, it may be remarked that we have not unfrequently used cinchona and the mineral acids in the treatment of typhus, and that too for the purposes for which they have been recommended, but so far, we have not seen any thing in their effects that entitles them to much if any consideration. When administered in doses sufficiently large to produce any effect on the disorder, this has generally been prejudicial.

Jamestown, O., July 1, 1844.

